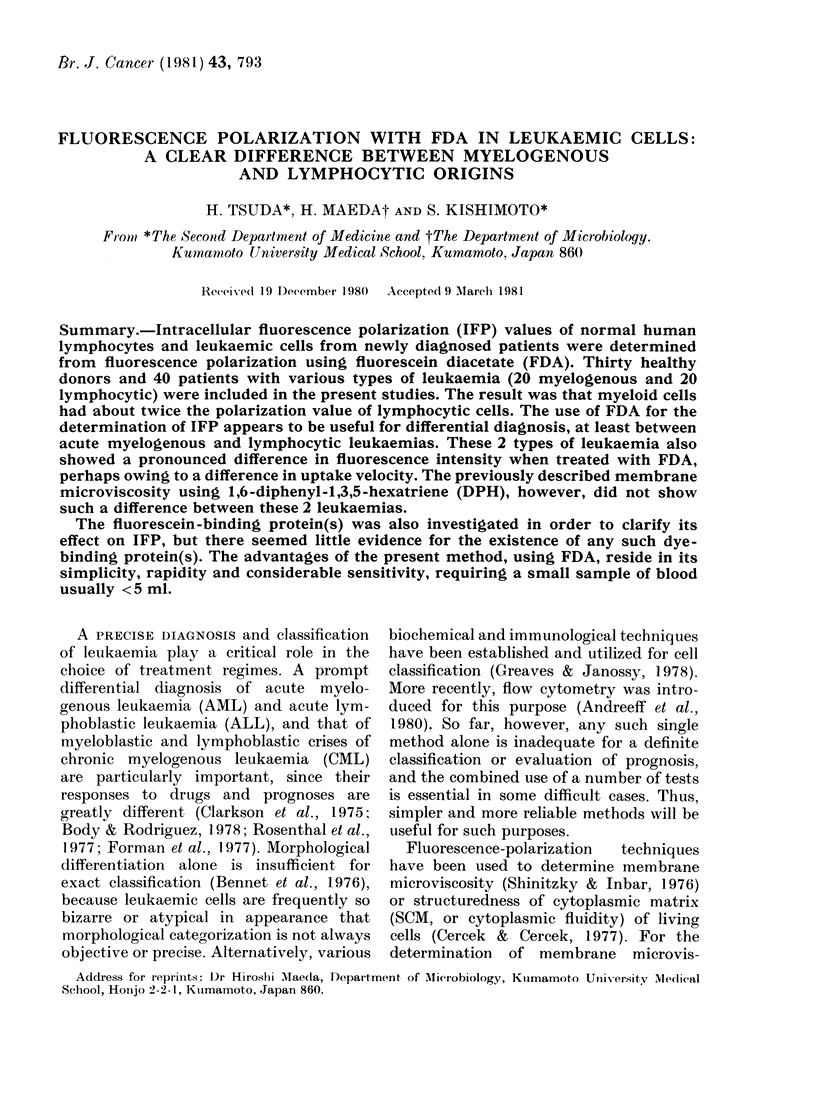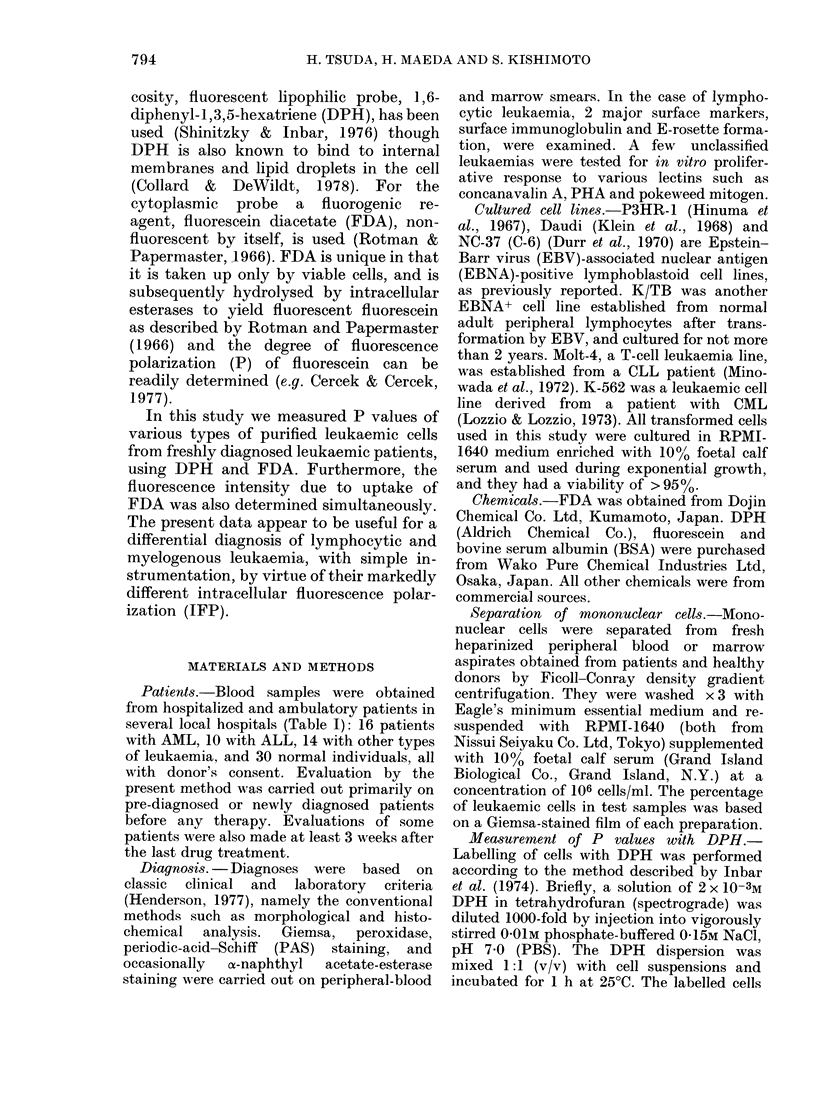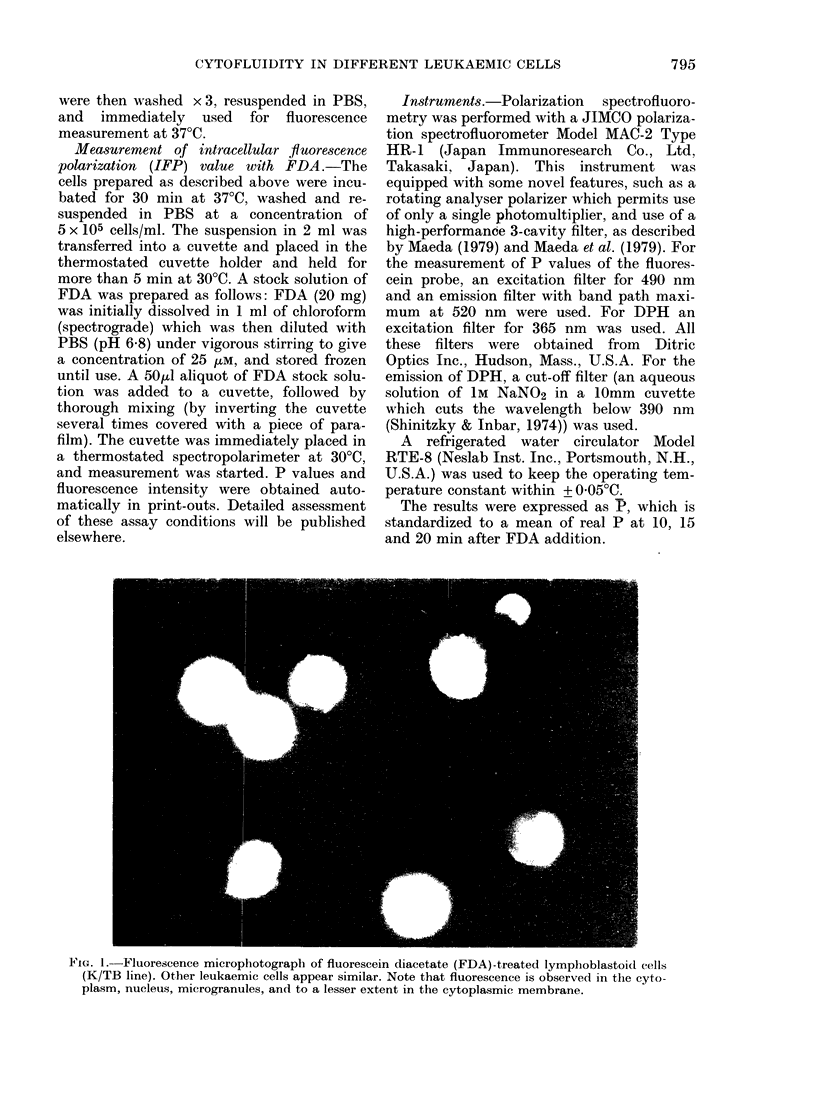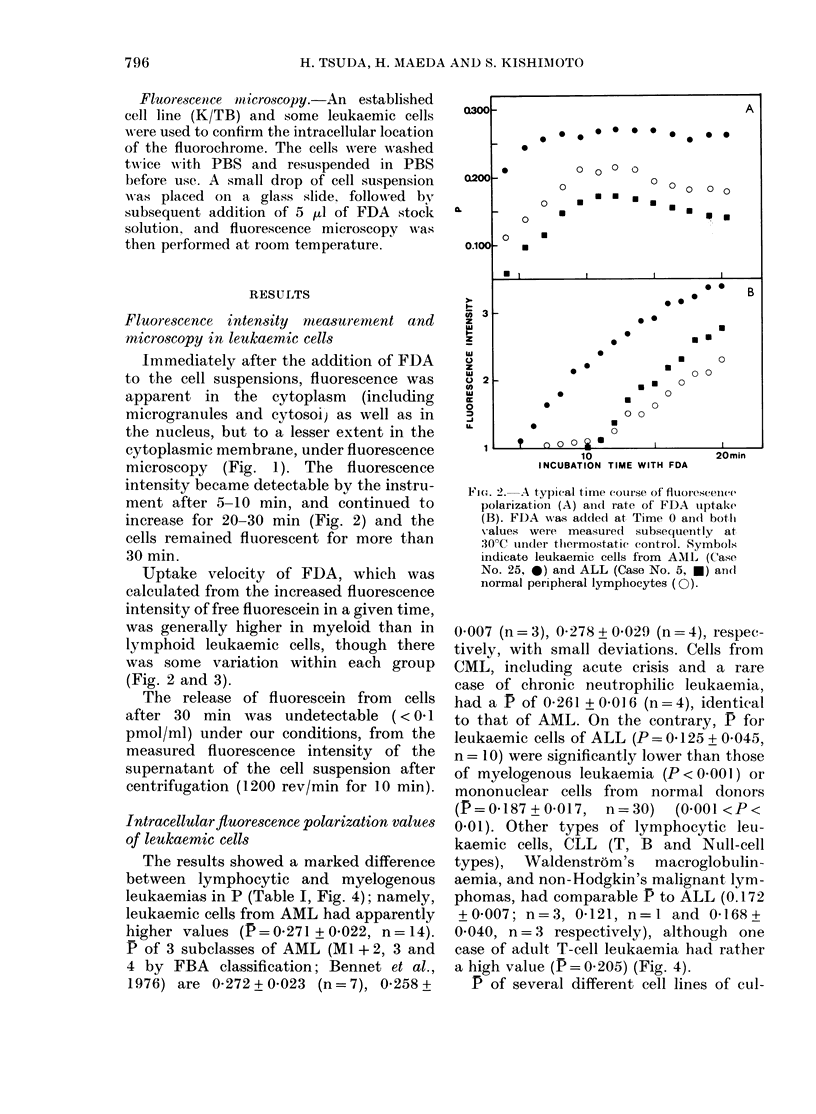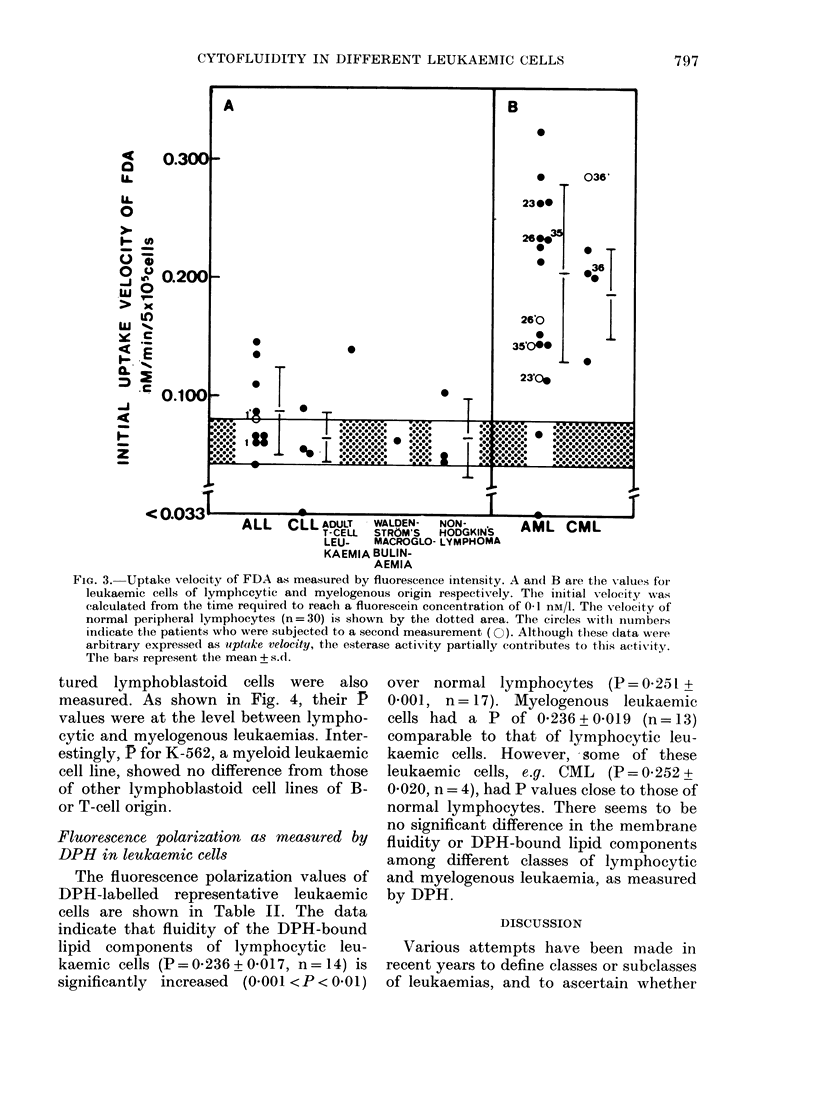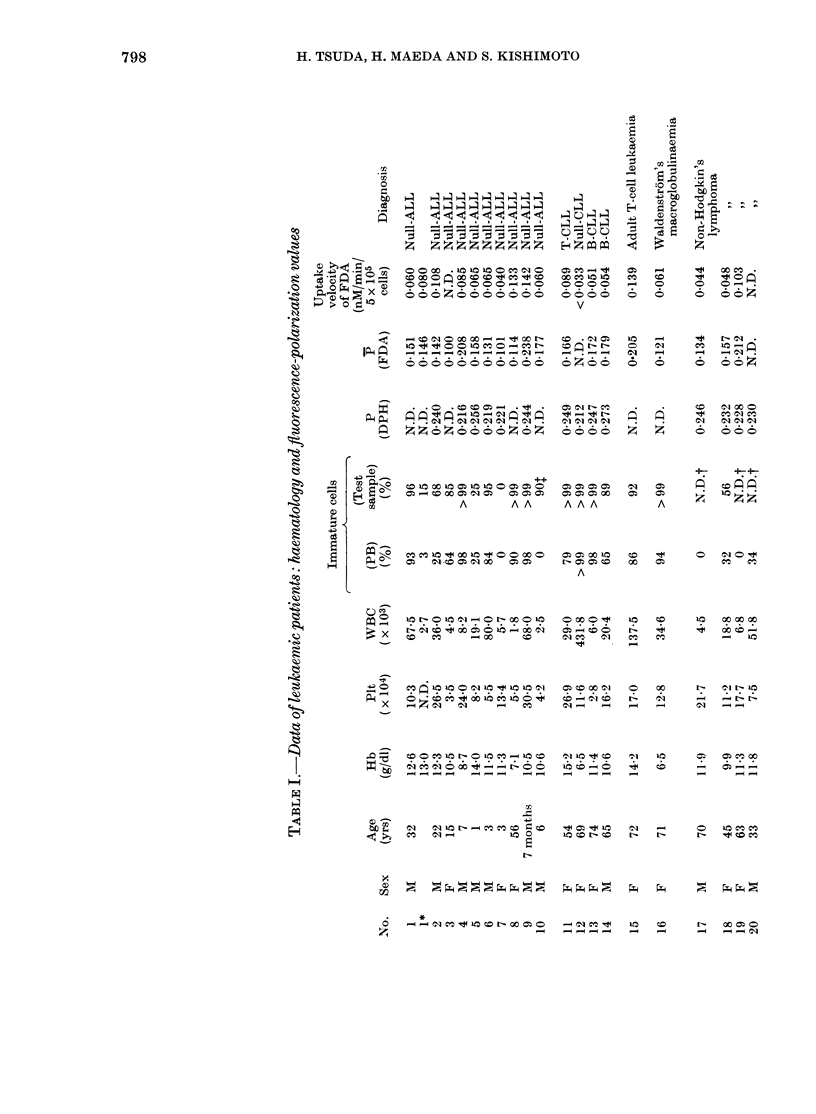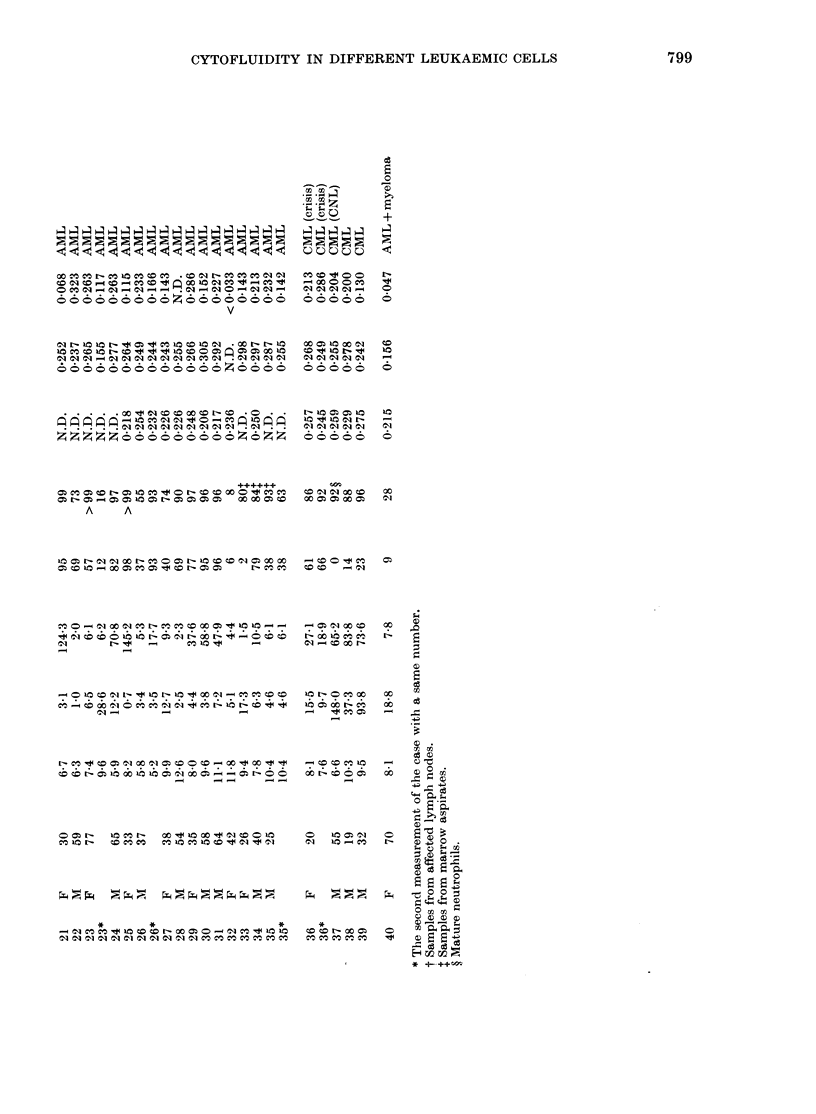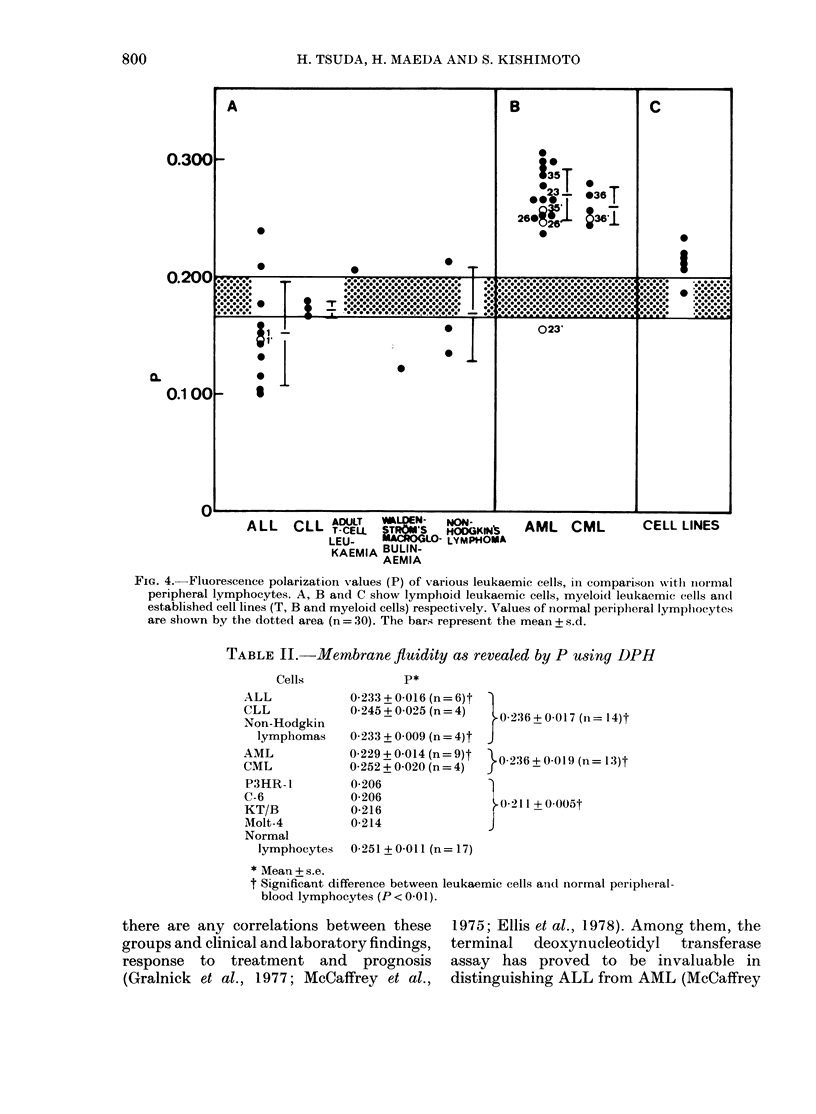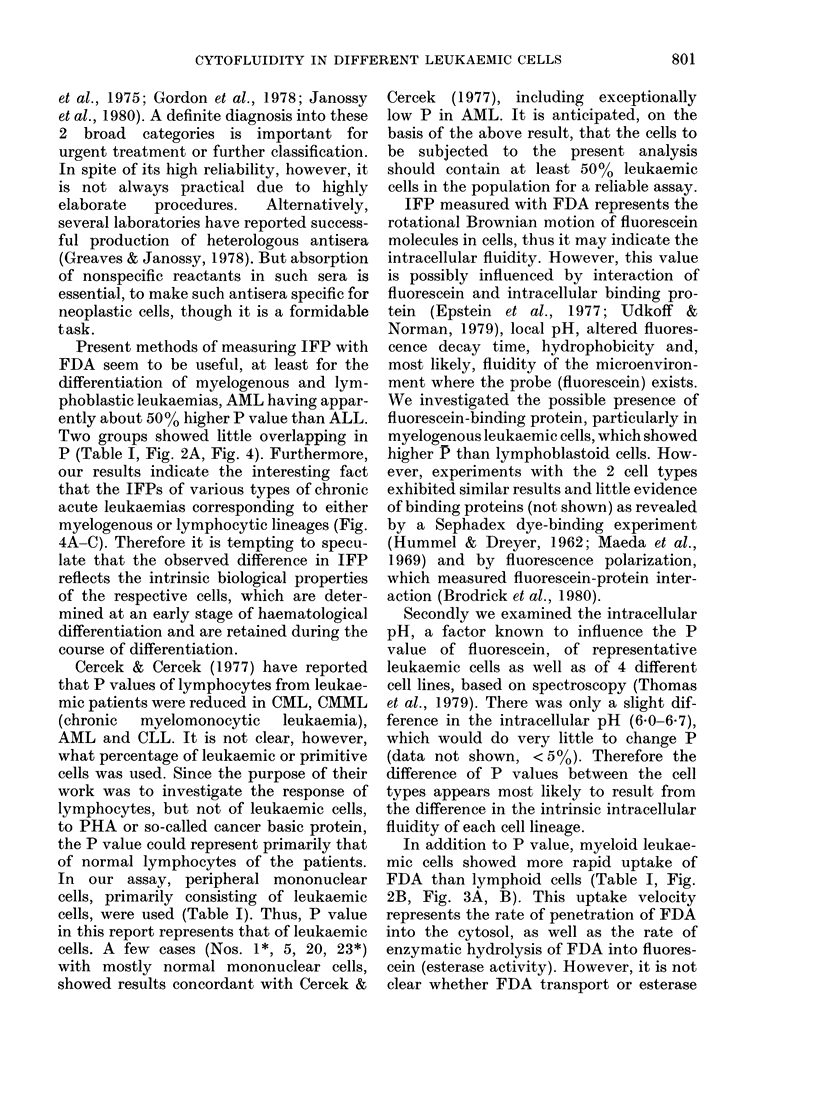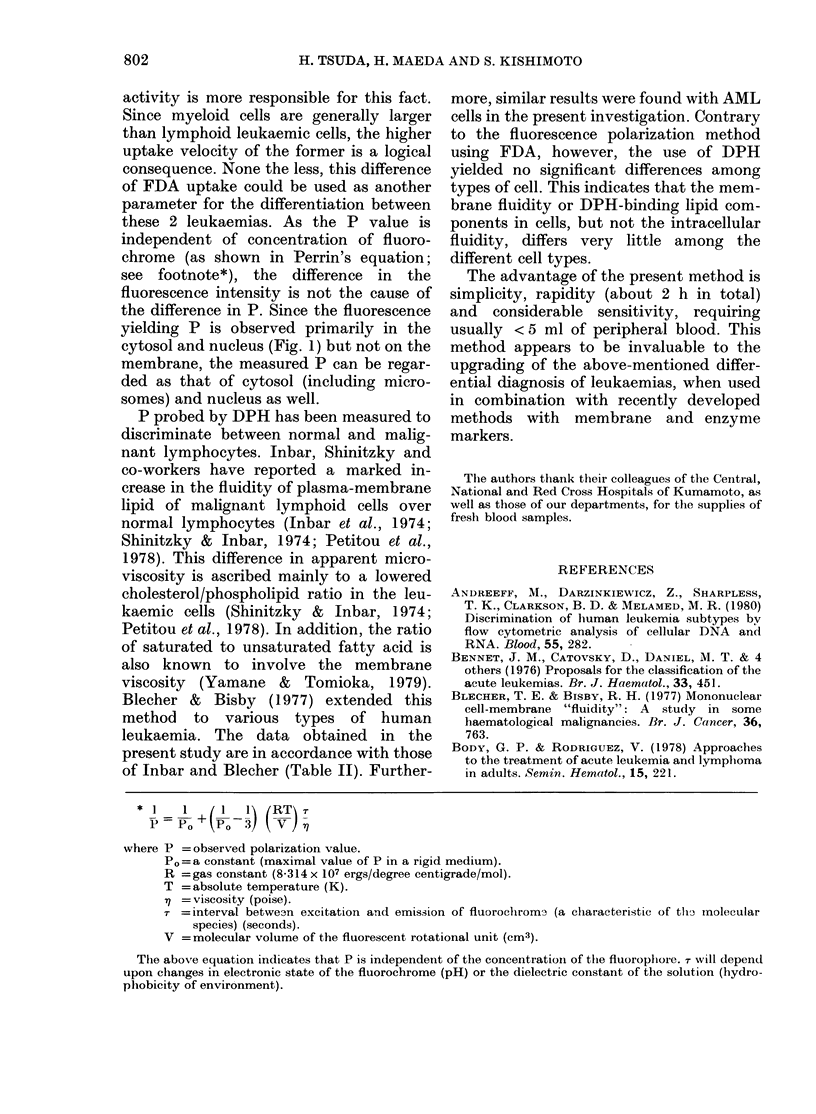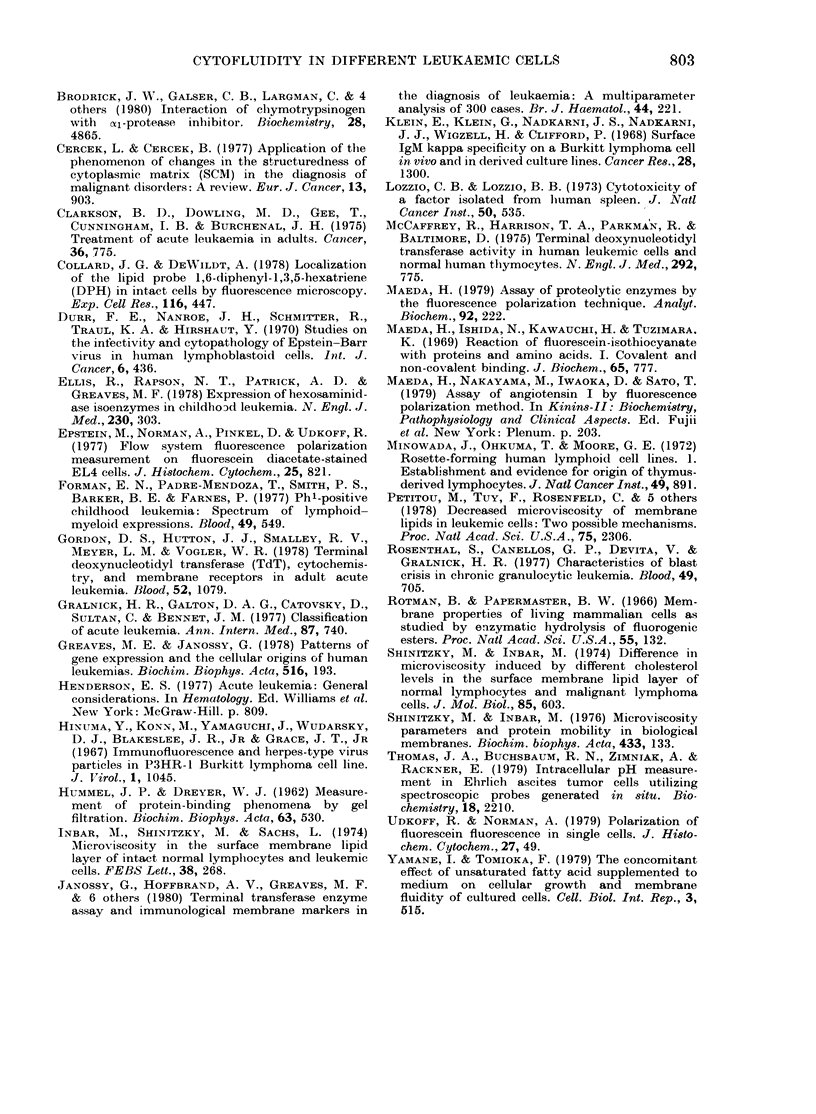# Fluorescence polarization with FDA in leukaemic cells: a clear difference between myelogenous and lymphocytic origins.

**DOI:** 10.1038/bjc.1981.117

**Published:** 1981-06

**Authors:** H. Tsuda, H. Maeda, S. Kishimoto

## Abstract

**Images:**


*Br. J. Cancer* (1981) **43**, 793

### FLUORESCENCE POLARIZATION WITH FDA IN LEUKAEMIC CELLS: A CLEAR DIFFERENCE BETWEEN MYELOGENOUS AND LYMPHOCYTIC ORIGINS

H. TSUDA*, H. MAEDA† AND S. KISHIMOTO*

*From *The Second Department of Medicine and †The Department of Microbiology, Kumamoto University Medical School, Kumamoto, Japan* 860

Received 19 December 1980 Accepted 9 March 1981

**Summary.—Intracellular fluorescence polarization (IFP) values of normal human lymphocytes and leukaemic cells from newly diagnosed patients were determined from fluorescence polarization using fluorescein diacetate (FDA). Thirty healthy donors and 40 patients with various types of leukaemia (20 myelogenous and 20 lymphocytic) were included in the present studies. The result was that myeloid cells had about twice the polarization value of lymphocytic cells. The use of FDA for the determination of IFP appears to be useful for differential diagnosis, at least between acute myelogenous and lymphocytic leukaemias. These 2 types of leukaemia also showed a pronounced difference in fluorescence intensity when treated with FDA, perhaps owing to a difference in uptake velocity. The previously described membrane microviscosity using 1,6-diphenyl-1,3,5-hexatriene (DPH), however, did not show such a difference between these 2 leukaemias.**

**The fluorescein-binding protein(s) was also investigated in order to clarify its effect on IFP, but there seemed little evidence for the existence of any such dye-binding protein(s). The advantages of the present method, using FDA, reside in its simplicity, rapidity and considerable sensitivity, requiring a small sample of blood usually <5 ml.**

A PRECISE DIAGNOSIS and classification of leukaemia play a critical role in the choice of treatment regimes. A prompt differential diagnosis of acute myelogenous leukaemia (AML) and acute lymphoblastic leukaemia (ALL), and that of myeloblastic and lymphoblastic crises of chronic myelogenous leukaemia (CML) are particularly important, since their responses to drugs and prognoses are greatly different (Clarkson *et al.*, 1975; Body & Rodriguez, 1978; Rosenthal *et al.*, 1977; Forman *et al.*, 1977). Morphological differentiation alone is insufficient for exact classification (Bennet *et al.*, 1976), because leukaemic cells are frequently so bizarre or atypical in appearance that morphological categorization is not always objective or precise. Alternatively, various biochemical and immunological techniques have been established and utilized for cell classification (Greaves & Janossy, 1978). More recently, flow cytometry was introduced for this purpose (Andreeff *et al.*, 1980). So far, however, any such single method alone is inadequate for a definite classification or evaluation of prognosis, and the combined use of a number of tests is essential in some difficult cases. Thus, simpler and more reliable methods will be useful for such purposes.

Fluorescence-polarization techniques have been used to determine membrane microviscosity (Shinitzky & Inbar, 1976) or structuredness of cytoplasmic matrix (SCM, or cytoplasmic fluidity) of living cells (Cercek & Cercek, 1977). For the determination of membrane microvis-

Address for reprints: Dr Hiroshi Maeda, Department of Microbiology, Kumamoto University Medical School, Honjo 2-2-1, Kumamoto, Japan 860.

cosity, fluorescent lipophilic probe, 1,6-diphenyl-1,3,5-hexatriene (DPH), has been used (Shinitzky & Inbar, 1976) though DPH is also known to bind to internal membranes and lipid droplets in the cell (Collard & DeWildt, 1978). For the cytoplasmic probe a fluorogenic reagent, fluorescein diacetate (FDA), non-fluorescent by itself, is used (Rotman & Papermaster, 1966). FDA is unique in that it is taken up only by viable cells, and is subsequently hydrolysed by intracellular esterases to yield fluorescent fluorescein as described by Rotman and Papermaster (1966) and the degree of fluorescence polarization (P) of fluorescein can be readily determined (*e.g.* Cercek & Cercek, 1977).

In this study we measured P values of various types of purified leukaemic cells from freshly diagnosed leukaemic patients, using DPH and FDA. Furthermore, the fluorescence intensity due to uptake of FDA was also determined simultaneously. The present data appear to be useful for a differential diagnosis of lymphocytic and myelogenous leukaemia, with simple instrumentation, by virtue of their markedly different intracellular fluorescence polarization (IFP).

### MATERIALS AND METHODS

*Patients.*—Blood samples were obtained from hospitalized and ambulatory patients in several local hospitals (Table I): 16 patients with AML, 10 with ALL, 14 with other types of leukaemia, and 30 normal individuals, all with donor's consent. Evaluation by the present method was carried out primarily on pre-diagnosed or newly diagnosed patients before any therapy. Evaluations of some patients were also made at least 3 weeks after the last drug treatment.

*Diagnosis.*—Diagnoses were based on classic clinical and laboratory criteria (Henderson, 1977), namely the conventional methods such as morphological and histochemical analysis. Giemsa, peroxidase, periodic-acid–Schiff (PAS) staining, and occasionally α-naphthyl acetate-esterase staining were carried out on peripheral-blood and marrow smears. In the case of lymphocytic leukaemia, 2 major surface markers, surface immunoglobulin and E-rosette formation, were examined. A few unclassified leukaemias were tested for *in vitro* proliferative response to various lectins such as concanavalin A, PHA and pokeweed mitogen.

*Cultured cell lines.*—P3HR-1 (Hinuma *et al.*, 1967), Daudi (Klein *et al.*, 1968) and NC-37 (C-6) (Durr *et al.*, 1970) are Epstein–Barr virus (EBV)-associated nuclear antigen (EBNA)-positive lymphoblastoid cell lines, as previously reported. K/TB was another $EBNA^+$ cell line established from normal adult peripheral lymphocytes after transformation by EBV, and cultured for not more than 2 years. Molt-4, a T-cell leukaemia line, was established from a CLL patient (Minowada *et al.*, 1972). K-562 was a leukaemic cell line derived from a patient with CML (Lozzio & Lozzio, 1973). All transformed cells used in this study were cultured in RPMI-1640 medium enriched with 10% foetal calf serum and used during exponential growth, and they had a viability of >95%.

*Chemicals.*—FDA was obtained from Dojin Chemical Co. Ltd, Kumamoto, Japan. DPH (Aldrich Chemical Co.), fluorescein and bovine serum albumin (BSA) were purchased from Wako Pure Chemical Industries Ltd, Osaka, Japan. All other chemicals were from commercial sources.

*Separation of mononuclear cells.*—Mononuclear cells were separated from fresh heparinized peripheral blood or marrow aspirates obtained from patients and healthy donors by Ficoll–Conray density gradient centrifugation. They were washed ×3 with Eagle's minimum essential medium and resuspended with RPMI-1640 (both from Nissui Seiyaku Co. Ltd, Tokyo) supplemented with 10% foetal calf serum (Grand Island Biological Co., Grand Island, N.Y.) at a concentration of $10^6$ cells/ml. The percentage of leukaemic cells in test samples was based on a Giemsa-stained film of each preparation.

*Measurement of P values with DPH.*—Labelling of cells with DPH was performed according to the method described by Inbar *et al.* (1974). Briefly, a solution of $2\times10^{-3}$M DPH in tetrahydrofuran (spectrograde) was diluted 1000-fold by injection into vigorously stirred 0·01M phosphate-buffered 0·15M NaCl, pH 7·0 (PBS). The DPH dispersion was mixed 1:1 (v/v) with cell suspensions and incubated for 1 h at 25°C. The labelled cells were then washed $\times 3$, resuspended in PBS, and immediately used for fluorescence measurement at 37°C.

*Measurement of intracellular fluorescence polarization (IFP) value with FDA*.—The cells prepared as described above were incubated for 30 min at 37°C, washed and resuspended in PBS at a concentration of $5 \times 10^5$ cells/ml. The suspension in 2 ml was transferred into a cuvette and placed in the thermostated cuvette holder and held for more than 5 min at 30°C. A stock solution of FDA was prepared as follows: FDA (20 mg) was initially dissolved in 1 ml of chloroform (spectrograde) which was then diluted with PBS (pH 6·8) under vigorous stirring to give a concentration of 25 $\mu$M, and stored frozen until use. A 50$\mu$l aliquot of FDA stock solution was added to a cuvette, followed by thorough mixing (by inverting the cuvette several times covered with a piece of parafilm). The cuvette was immediately placed in a thermostated spectropolarimeter at 30°C, and measurement was started. P values and fluorescence intensity were obtained automatically in print-outs. Detailed assessment of these assay conditions will be published elsewhere.

*Instruments*.—Polarization spectrofluorometry was performed with a JIMCO polarization spectrofluorometer Model MAC-2 Type HR-1 (Japan Immunoresearch Co., Ltd, Takasaki, Japan). This instrument was equipped with some novel features, such as a rotating analyser polarizer which permits use of only a single photomultiplier, and use of a high-performance 3-cavity filter, as described by Maeda (1979) and Maeda *et al.* (1979). For the measurement of P values of the fluorescein probe, an excitation filter for 490 nm and an emission filter with band path maximum at 520 nm were used. For DPH an excitation filter for 365 nm was used. All these filters were obtained from Ditric Optics Inc., Hudson, Mass., U.S.A. For the emission of DPH, a cut-off filter (an aqueous solution of 1M $NaNO_2$ in a 10mm cuvette which cuts the wavelength below 390 nm (Shinitzky & Inbar, 1974)) was used.

A refrigerated water circulator Model RTE-8 (Neslab Inst. Inc., Portsmouth, N.H., U.S.A.) was used to keep the operating temperature constant within $\pm 0{\cdot}05$°C.

The results were expressed as $\bar{P}$, which is standardized to a mean of real P at 10, 15 and 20 min after FDA addition.

FIG. 1.—Fluorescence microphotograph of fluorescein diacetate (FDA)-treated lymphoblastoid cells (K/TB line). Other leukaemic cells appear similar. Note that fluorescence is observed in the cytoplasm, nucleus, microgranules, and to a lesser extent in the cytoplasmic membrane.

*Fluorescence microscopy.*—An established cell line (K/TB) and some leukaemic cells were used to confirm the intracellular location of the fluorochrome. The cells were washed twice with PBS and resuspended in PBS before use. A small drop of cell suspension was placed on a glass slide, followed by subsequent addition of 5 $\mu$l of FDA stock solution, and fluorescence microscopy was then performed at room temperature.

### RESULTS

### *Fluorescence intensity measurement and microscopy in leukaemic cells*

Immediately after the addition of FDA to the cell suspensions, fluorescence was apparent in the cytoplasm (including microgranules and cytosol) as well as in the nucleus, but to a lesser extent in the cytoplasmic membrane, under fluorescence microscopy (Fig. 1). The fluorescence intensity became detectable by the instrument after 5–10 min, and continued to increase for 20–30 min (Fig. 2) and the cells remained fluorescent for more than 30 min.

Uptake velocity of FDA, which was calculated from the increased fluorescence intensity of free fluorescein in a given time, was generally higher in myeloid than in lymphoid leukaemic cells, though there was some variation within each group (Fig. 2 and 3).

The release of fluorescein from cells after 30 min was undetectable ($<0{\cdot}1$ pmol/ml) under our conditions, from the measured fluorescence intensity of the supernatant of the cell suspension after centrifugation (1200 rev/min for 10 min).

### *Intracellular fluorescence polarization values of leukaemic cells*

The results showed a marked difference between lymphocytic and myelogenous leukaemias in P (Table I, Fig. 4); namely, leukaemic cells from AML had apparently higher values ($\bar{P} = 0{\cdot}271 \pm 0{\cdot}022$, n = 14). $\bar{P}$ of 3 subclasses of AML (M1 + 2, 3 and 4 by FBA classification; Bennet *et al.*, 1976) are $0{\cdot}272 \pm 0{\cdot}023$ (n = 7), $0{\cdot}258 \pm 0{\cdot}007$ (n = 3), $0{\cdot}278 \pm 0{\cdot}029$ (n = 4), respectively, with small deviations. Cells from CML, including acute crisis and a rare case of chronic neutrophilic leukaemia, had a $\bar{P}$ of $0{\cdot}261 \pm 0{\cdot}016$ (n = 4), identical to that of AML. On the contrary, $\bar{P}$ for leukaemic cells of ALL ($P = 0{\cdot}125 \pm 0{\cdot}045$, n = 10) were significantly lower than those of myelogenous leukaemia ($P < 0{\cdot}001$) or mononuclear cells from normal donors ($\bar{P} = 0{\cdot}187 \pm 0{\cdot}017$, n = 30) ($0{\cdot}001 < P < 0{\cdot}01$). Other types of lymphocytic leukaemic cells, CLL (T, B and Null-cell types), Waldenström's macroglobulinaemia, and non-Hodgkin's malignant lymphomas, had comparable $\bar{P}$ to ALL (0.172 $\pm 0{\cdot}007$; n = 3, 0·121, n = 1 and $0{\cdot}168 \pm 0{\cdot}040$, n = 3 respectively), although one case of adult T-cell leukaemia had rather a high value ($\bar{P} = 0{\cdot}205$) (Fig. 4).

FIG. 2.—A typical time course of fluorescence polarization (A) and rate of FDA uptake (B). FDA was added at Time 0 and both values were measured subsequently at 30°C under thermostatic control. Symbols indicate leukaemic cells from AML (Case No. 25, ●) and ALL (Case No. 5, ■) and normal peripheral lymphocytes (○).

$\bar{P}$ of several different cell lines of cul-

FIG. 3.—Uptake velocity of FDA as measured by fluorescence intensity. A and B are the values for leukaemic cells of lymphocytic and myelogenous origin respectively. The initial velocity was calculated from the time required to reach a fluorescein concentration of 0·1 nM/l. The velocity of normal peripheral lymphocytes (n = 30) is shown by the dotted area. The circles with numbers indicate the patients who were subjected to a second measurement (○). Although these data were arbitrary expressed as *uptake velocity*, the esterase activity partially contributes to this activity. The bars represent the mean ± s.d.

tured lymphoblastoid cells were also measured. As shown in Fig. 4, their $\bar{P}$ values were at the level between lymphocytic and myelogenous leukaemias. Interestingly, $\bar{P}$ for K-562, a myeloid leukaemic cell line, showed no difference from those of other lymphoblastoid cell lines of B- or T-cell origin.

*Fluorescence polarization as measured by DPH in leukaemic cells*

The fluorescence polarization values of DPH-labelled representative leukaemic cells are shown in Table II. The data indicate that fluidity of the DPH-bound lipid components of lymphocytic leukaemic cells (P = 0·236 ± 0·017, n = 14) is significantly increased (0·001 < $P$ < 0·01) over normal lymphocytes (P = 0·251 ± 0·001, n = 17). Myelogenous leukaemic cells had a P of 0·236 ± 0·019 (n = 13) comparable to that of lymphocytic leukaemic cells. However, some of these leukaemic cells, *e.g.* CML (P = 0·252 ± 0·020, n = 4), had P values close to those of normal lymphocytes. There seems to be no significant difference in the membrane fluidity or DPH-bound lipid components among different classes of lymphocytic and myelogenous leukaemia, as measured by DPH.

### DISCUSSION

Various attempts have been made in recent years to define classes or subclasses of leukaemias, and to ascertain whether

TABLE I.—*Data of leukaemic patients: haematology and fluorescence-polarization values*

| No. | Sex | Age (yrs) | Hb (g/dl) | Plt (× $10^4$) | WBC (× $10^3$) | Immature cells (PB) (%) | Immature cells (Test sample) (%) | P (DPH) | $\bar{P}$ (FDA) | Uptake velocity of FDA (nM/min/ 5 × $10^5$ cells) | Diagnosis |
|---|---|---|---|---|---|---|---|---|---|---|---|
| 1 | M | 32 | 12·6 | 10·3 | 67·5 | 93 | 96 | N.D. | 0·151 | 0·060 | Null-ALL |
| 1* | | | 13·0 | N.D. | 2·7 | 3 | 15 | N.D. | 0·146 | 0·080 | |
| 2 | M | 22 | 12·3 | 26·5 | 36·0 | 25 | 68 | 0·240 | 0·142 | 0·108 | Null-ALL |
| 3 | F | 15 | 10·5 | 3·5 | 4·5 | 64 | 85 | N.D. | 0·100 | N.D. | Null-ALL |
| 4 | M | 7 | 8·7 | 24·0 | 8·2 | 98 | > 99 | 0·216 | 0·208 | 0·085 | Null-ALL |
| 5 | M | 1 | 14·0 | 8·2 | 19·1 | 25 | 25 | 0·256 | 0·158 | 0·065 | Null-ALL |
| 6 | M | 3 | 11·5 | 5·5 | 80·0 | 84 | 95 | 0·219 | 0·131 | 0·065 | Null-ALL |
| 7 | F | 3 | 11·3 | 13·4 | 5·7 | 0 | 0 | 0·221 | 0·101 | 0·040 | Null-ALL |
| 8 | F | 56 | 7·1 | 5·5 | 1·8 | 90 | > 99 | N.D. | 0·114 | 0·133 | Null-ALL |
| 9 | M | 7 months | 10·5 | 30·5 | 68·0 | 98 | > 99 | 0·244 | 0·238 | 0·142 | Null-ALL |
| 10 | M | 6 | 10·6 | 4·2 | 2·5 | 0 | 90‡ | N.D. | 0·177 | 0·060 | Null-ALL |
| 11 | F | 54 | 15·2 | 26·9 | 29·0 | 79 | > 99 | 0·249 | 0·166 | 0·089 | T-CLL |
| 12 | F | 69 | 6·5 | 11·6 | 431·8 | > 99 | > 99 | 0·212 | N.D. | < 0·033 | Null-CLL |
| 13 | F | 74 | 11·4 | 2·8 | 6·0 | 98 | > 99 | 0·247 | 0·172 | 0·051 | B-CLL |
| 14 | M | 65 | 10·6 | 16·2 | 20·4 | 65 | 89 | 0·273 | 0·179 | 0·054 | B-CLL |
| 15 | F | 72 | 14·2 | 17·0 | 137·5 | 86 | 92 | N.D. | 0·205 | 0·139 | Adult T-cell leukaemia |
| 16 | F | 71 | 6·5 | 12·8 | 34·6 | 94 | > 99 | N.D. | 0·121 | 0·061 | Waldenström's macroglobulinaemia |
| 17 | M | 70 | 11·9 | 21·7 | 4·5 | 0 | N.D.† | 0·246 | 0·134 | 0·044 | Non-Hodgkin's lymphoma |
| 18 | F | 45 | 9·9 | 11·2 | 18·8 | 32 | 56 | 0·232 | 0·157 | 0·048 | ,, |
| 19 | F | 63 | 11·3 | 17·7 | 6·8 | 0 | N.D.† | 0·228 | 0·212 | 0·103 | ,, |
| 20 | M | 33 | 11·8 | 7·5 | 51·8 | 34 | N.D.† | 0·230 | N.D. | N.D. | ,, |

| | | | | | | | | | | | |
|---|---|---|---|---|---|---|---|---|---|---|---|
| 21 | F | 30 | 6·7 | 3·1 | 124·3 | 95 | 99 | N.D. | 0·252 | 0·068 | AML |
| 22 | M | 59 | 6·3 | 1·0 | 2·0 | 69 | 73 | N.D. | 0·237 | 0·323 | AML |
| 23 | F | 77 | 7·4 | 6·5 | 6·1 | 57 | > 99 | N.D. | 0·265 | 0·263 | AML |
| 23* | | | 9·6 | 28·6 | 6·2 | 12 | 16 | N.D. | 0·155 | 0·117 | AML |
| 24 | M | 65 | 5·9 | 12·2 | 70·8 | 82 | 97 | N.D. | 0·277 | 0·263 | AML |
| 25 | F | 33 | 8·2 | 0·7 | 145·2 | 98 | > 99 | 0·218 | 0·264 | 0·115 | AML |
| 26 | M | 37 | 5·8 | 3·4 | 5·3 | 37 | 55 | 0·254 | 0·249 | 0·233 | AML |
| 26* | | | 5·2 | 3·5 | 17·7 | 93 | 93 | 0·232 | 0·244 | 0·166 | AML |
| 27 | F | 38 | 9·9 | 12·7 | 9·3 | 40 | 74 | 0·226 | 0·243 | 0·143 | AML |
| 28 | M | 54 | 12·6 | 2·5 | 2·3 | 69 | 90 | 0·226 | 0·255 | N.D. | AML |
| 29 | F | 35 | 8·0 | 4·4 | 37·6 | 77 | 97 | 0·248 | 0·266 | 0·286 | AML |
| 30 | M | 58 | 9·6 | 3·8 | 58·8 | 95 | 96 | 0·206 | 0·305 | 0·152 | AML |
| 31 | M | 64 | 11·1 | 7·2 | 47·9 | 96 | 96 | 0·217 | 0·292 | 0·227 | AML |
| 32 | F | 42 | 11·8 | 5·1 | 4·4 | 6 | 8 | 0·236 | N.D. | < 0·033 | AML |
| 33 | F | 26 | 9·4 | 17·3 | 1·5 | 2 | 80‡ | N.D. | 0·298 | 0·143 | AML |
| 34 | M | 40 | 7·8 | 6·3 | 10·5 | 79 | 84‡ | 0·250 | 0·297 | 0·213 | AML |
| 35 | M | 25 | 10·4 | 4·6 | 6·1 | 38 | 93‡ | N.D. | 0·287 | 0·232 | AML |
| 35* | | | 10·4 | 4·6 | 6·1 | 38 | 63 | N.D. | 0·255 | 0·142 | AML |
| 36 | F | 20 | 8·1 | 15·5 | 27·1 | 61 | 86 | 0·257 | 0·268 | 0·213 | CML (crisis) |
| 36* | | | 7·6 | 9·7 | 18·9 | 66 | 92 | 0·245 | 0·249 | 0·286 | CML (crisis) |
| 37 | M | 55 | 6·6 | 148·0 | 65·2 | 0 | 92§ | 0·259 | 0·255 | 0·204 | CML (CNL) |
| 38 | M | 19 | 10·3 | 37·3 | 83·8 | 14 | 88 | 0·229 | 0·278 | 0·200 | CML |
| 39 | M | 32 | 9·5 | 93·8 | 73·6 | 23 | 96 | 0·275 | 0·242 | 0·130 | CML |
| 40 | F | 70 | 8·1 | 18·8 | 7·8 | 9 | 28 | 0·215 | 0·156 | 0·047 | AML + myeloma |

* The second measurement of the case with a same number.

† Samples from affected lymph nodes.

‡ Samples from marrow aspirates.

§ Mature neutrophils.

FIG. 4.—Fluorescence polarization values (P) of various leukaemic cells, in comparison with normal peripheral lymphocytes. A, B and C show lymphoid leukaemic cells, myeloid leukaemic cells and established cell lines (T, B and myeloid cells) respectively. Values of normal peripheral lymphocytes are shown by the dotted area (n = 30). The bars represent the mean ± s.d.

TABLE II.—*Membrane fluidity as revealed by P using DPH*

| Cells | P* | |
|---|---|---|
| ALL | 0·233 ± 0·016 (n = 6)† | 0·236 ± 0·017 (n = 14)† |
| CLL | 0·245 ± 0·025 (n = 4) | |
| Non-Hodgkin lymphomas | 0·233 ± 0·009 (n = 4)† | |
| AML | 0·229 ± 0·014 (n = 9)† | 0·236 ± 0·019 (n = 13)† |
| CML | 0·252 ± 0·020 (n = 4) | |
| P3HR-1 | 0·206 | 0·211 ± 0·005† |
| C-6 | 0·206 | |
| KT/B | 0·216 | |
| Molt-4 | 0·214 | |
| Normal lymphocytes | 0·251 ± 0·011 (n = 17) | |

* Mean ± s.e.
† Significant difference between leukaemic cells and normal peripheral-blood lymphocytes ($P < 0·01$).

there are any correlations between these groups and clinical and laboratory findings, response to treatment and prognosis (Gralnick *et al.*, 1977; McCaffrey *et al.*, 1975; Ellis *et al.*, 1978). Among them, the terminal deoxynucleotidyl transferase assay has proved to be invaluable in distinguishing ALL from AML (McCaffrey

*et al.*, 1975; Gordon *et al.*, 1978; Janossy *et al.*, 1980). A definite diagnosis into these 2 broad categories is important for urgent treatment or further classification. In spite of its high reliability, however, it is not always practical due to highly elaborate procedures. Alternatively, several laboratories have reported successful production of heterologous antisera (Greaves & Janossy, 1978). But absorption of nonspecific reactants in such sera is essential, to make such antisera specific for neoplastic cells, though it is a formidable task.

Present methods of measuring IFP with FDA seem to be useful, at least for the differentiation of myelogenous and lymphoblastic leukaemias, AML having apparently about 50% higher P value than ALL. Two groups showed little overlapping in P (Table I, Fig. 2A, Fig. 4). Furthermore, our results indicate the interesting fact that the IFPs of various types of chronic acute leukaemias corresponding to either myelogenous or lymphocytic lineages (Fig. 4A–C). Therefore it is tempting to speculate that the observed difference in IFP reflects the intrinsic biological properties of the respective cells, which are determined at an early stage of haematological differentiation and are retained during the course of differentiation.

Cercek & Cercek (1977) have reported that P values of lymphocytes from leukaemic patients were reduced in CML, CMML (chronic myelomonocytic leukaemia), AML and CLL. It is not clear, however, what percentage of leukaemic or primitive cells was used. Since the purpose of their work was to investigate the response of lymphocytes, but not of leukaemic cells, to PHA or so-called cancer basic protein, the P value could represent primarily that of normal lymphocytes of the patients. In our assay, peripheral mononuclear cells, primarily consisting of leukaemic cells, were used (Table I). Thus, P value in this report represents that of leukaemic cells. A few cases (Nos. 1*, 5, 20, 23*) with mostly normal mononuclear cells, showed results concordant with Cercek & Cercek (1977), including exceptionally low P in AML. It is anticipated, on the basis of the above result, that the cells to be subjected to the present analysis should contain at least 50% leukaemic cells in the population for a reliable assay.

IFP measured with FDA represents the rotational Brownian motion of fluorescein molecules in cells, thus it may indicate the intracellular fluidity. However, this value is possibly influenced by interaction of fluorescein and intracellular binding protein (Epstein *et al.*, 1977; Udkoff & Norman, 1979), local pH, altered fluorescence decay time, hydrophobicity and, most likely, fluidity of the microenvironment where the probe (fluorescein) exists. We investigated the possible presence of fluorescein-binding protein, particularly in myelogenous leukaemic cells, which showed higher $\bar{P}$ than lymphoblastoid cells. However, experiments with the 2 cell types exhibited similar results and little evidence of binding proteins (not shown) as revealed by a Sephadex dye-binding experiment (Hummel & Dreyer, 1962; Maeda *et al.*, 1969) and by fluorescence polarization, which measured fluorescein-protein interaction (Brodrick *et al.*, 1980).

Secondly we examined the intracellular pH, a factor known to influence the P value of fluorescein, of representative leukaemic cells as well as of 4 different cell lines, based on spectroscopy (Thomas *et al.*, 1979). There was only a slight difference in the intracellular pH (6·0–6·7), which would do very little to change P (data not shown, $<5\%$). Therefore the difference of P values between the cell types appears most likely to result from the difference in the intrinsic intracellular fluidity of each cell lineage.

In addition to P value, myeloid leukaemic cells showed more rapid uptake of FDA than lymphoid cells (Table I, Fig. 2B, Fig. 3A, B). This uptake velocity represents the rate of penetration of FDA into the cytosol, as well as the rate of enzymatic hydrolysis of FDA into fluorescein (esterase activity). However, it is not clear whether FDA transport or esterase activity is more responsible for this fact. Since myeloid cells are generally larger than lymphoid leukaemic cells, the higher uptake velocity of the former is a logical consequence. None the less, this difference of FDA uptake could be used as another parameter for the differentiation between these 2 leukaemias. As the P value is independent of concentration of fluorochrome (as shown in Perrin's equation; see footnote*), the difference in the fluorescence intensity is not the cause of the difference in P. Since the fluorescence yielding P is observed primarily in the cytosol and nucleus (Fig. 1) but not on the membrane, the measured P can be regarded as that of cytosol (including microsomes) and nucleus as well.

P probed by DPH has been measured to discriminate between normal and malignant lymphocytes. Inbar, Shinitzky and co-workers have reported a marked increase in the fluidity of plasma-membrane lipid of malignant lymphoid cells over normal lymphocytes (Inbar *et al.*, 1974; Shinitzky & Inbar, 1974; Petitou *et al.*, 1978). This difference in apparent microviscosity is ascribed mainly to a lowered cholesterol/phospholipid ratio in the leukaemic cells (Shinitzky & Inbar, 1974; Petitou *et al.*, 1978). In addition, the ratio of saturated to unsaturated fatty acid is also known to involve the membrane viscosity (Yamane & Tomioka, 1979). Blecher & Bisby (1977) extended this method to various types of human leukaemia. The data obtained in the present study are in accordance with those of Inbar and Blecher (Table II). Furthermore, similar results were found with AML cells in the present investigation. Contrary to the fluorescence polarization method using FDA, however, the use of DPH yielded no significant differences among types of cell. This indicates that the membrane fluidity or DPH-binding lipid components in cells, but not the intracellular fluidity, differs very little among the different cell types.

The advantage of the present method is simplicity, rapidity (about 2 h in total) and considerable sensitivity, requiring usually < 5 ml of peripheral blood. This method appears to be invaluable to the upgrading of the above-mentioned differential diagnosis of leukaemias, when used in combination with recently developed methods with membrane and enzyme markers.

The authors thank their colleagues of the Central, National and Red Cross Hospitals of Kumamoto, as well as those of our departments, for the supplies of fresh blood samples.

### REFERENCES

ANDREEFF, M., DARZINKIEWICZ, Z., SHARPLESS, T. K., CLARKSON, B. D. & MELAMED, M. R. (1980) Discrimination of human leukemia subtypes by flow cytometric analysis of cellular DNA and RNA. *Blood*, **55**, 282.

BENNET, J. M., CATOVSKY, D., DANIEL, M. T. & 4 others (1976) Proposals for the classification of the acute leukemias. *Br. J. Haematol.*, **33**, 451.

BLECHER, T. E. & BISBY, R. H. (1977) Mononuclear cell-membrane "fluidity": A study in some haematological malignancies. *Br. J. Cancer*, **36**, 763.

BODY, G. P. & RODRIGUEZ, V. (1978) Approaches to the treatment of acute leukemia and lymphoma in adults. *Semin. Hematol.*, **15**, 221.

* $$\frac{1}{P} = \frac{1}{P_0} + \left(\frac{1}{P_0} - \frac{1}{3}\right)\left(\frac{RT}{V}\right)\frac{\tau}{\eta}$$

where P = observed polarization value.
$P_0$ = a constant (maximal value of P in a rigid medium).
R = gas constant ($8{\cdot}314 \times 10^7$ ergs/degree centigrade/mol).
T = absolute temperature (K).
$\eta$ = viscosity (poise).
$\tau$ = interval between excitation and emission of fluorochrome (a characteristic of the molecular species) (seconds).
V = molecular volume of the fluorescent rotational unit ($cm^3$).

The above equation indicates that P is independent of the concentration of the fluorophore. $\tau$ will depend upon changes in electronic state of the fluorochrome (pH) or the dielectric constant of the solution (hydrophobicity of environment).

Brodrick, J. W., Galser, C. B., Largman, C. & 4 others (1980) Interaction of chymotrypsinogen with $\alpha_1$-protease inhibitor. *Biochemistry*, **28**, 4865.

Cercek, L. & Cercek, B. (1977) Application of the phenomenon of changes in the structuredness of cytoplasmic matrix (SCM) in the diagnosis of malignant disorders: A review. *Eur. J. Cancer*, **13**, 903.

Clarkson, B. D., Dowling, M. D., Gee, T., Cunningham, I. B. & Burchenal, J. H. (1975) Treatment of acute leukaemia in adults. *Cancer*, **36**, 775.

Collard, J. G. & DeWildt, A. (1978) Localization of the lipid probe 1,6-diphenyl-1,3,5-hexatriene (DPH) in intact cells by fluorescence microscopy. *Exp. Cell Res.*, **116**, 447.

Durr, F. E., Nanroe, J. H., Schmitter, R., Traul, K. A. & Hirshaut, Y. (1970) Studies on the infectivity and cytopathology of Epstein–Barr virus in human lymphoblastoid cells. *Int. J. Cancer*, **6**, 436.

Ellis, R., Rapson, N. T., Patrick, A. D. & Greaves, M. F. (1978) Expression of hexosaminidase isoenzymes in childhood leukemia. *N. Engl. J. Med.*, **230**, 303.

Epstein, M., Norman, A., Pinkel, D. & Udkoff, R. (1977) Flow system fluorescence polarization measurement on fluorescein diacetate-stained EL4 cells. *J. Histochem. Cytochem.*, **25**, 821.

Forman, E. N., Padre-Mendoza, T., Smith, P. S., Barker, B. E. & Farnes, P. (1977) Ph[1]-positive childhood leukemia: Spectrum of lymphoid–myeloid expressions. *Blood*, **49**, 549.

Gordon, D. S., Hutton, J. J., Smalley, R. V., Meyer, L. M. & Vogler, W. R. (1978) Terminal deoxynucleotidyl transferase (TdT), cytochemistry, and membrane receptors in adult acute leukemia. *Blood*, **52**, 1079.

Gralnick, H. R., Galton, D. A. G., Catovsky, D., Sultan, C. & Bennet, J. M. (1977) Classification of acute leukemia. *Ann. Intern. Med.*, **87**, 740.

Greaves, M. E. & Janossy, G. (1978) Patterns of gene expression and the cellular origins of human leukemias. *Biochim. Biophys. Acta*, **516**, 193.

Henderson, E. S. (1977) Acute leukemia: General considerations. In *Hematology*. Ed. Williams *et al.* New York: McGraw-Hill. p. 809.

Hinuma, Y., Konn, M., Yamaguchi, J., Wudarsky, D. J., Blakeslee, J. R., Jr & Grace, J. T., Jr (1967) Immunofluorescence and herpes-type virus particles in P3HR-1 Burkitt lymphoma cell line. *J. Virol.*, **1**, 1045.

Hummel, J. P. & Dreyer, W. J. (1962) Measurement of protein-binding phenomena by gel filtration. *Biochim. Biophys. Acta*, **63**, 530.

Inbar, M., Shinitzky, M. & Sachs, L. (1974) Microviscosity in the surface membrane lipid layer of intact normal lymphocytes and leukemic cells. *FEBS Lett.*, **38**, 268.

Janossy, G., Hoffbrand, A. V., Greaves, M. F. & 6 others (1980) Terminal transferase enzyme assay and immunological membrane markers in the diagnosis of leukaemia: A multiparameter analysis of 300 cases. *Br. J. Haematol.*, **44**, 221.

Klein, E., Klein, G., Nadkarni, J. S., Nadkarni, J. J., Wigzell, H. & Clifford, P. (1968) Surface IgM kappa specificity on a Burkitt lymphoma cell *in vivo* and in derived culture lines. *Cancer Res.*, **28**, 1300.

Lozzio, C. B. & Lozzio, B. B. (1973) Cytotoxicity of a factor isolated from human spleen. *J. Natl Cancer Inst.*, **50**, 535.

McCaffrey, R., Harrison, T. A., Parkman, R. & Baltimore, D. (1975) Terminal deoxynucleotidyl transferase activity in human leukemic cells and normal human thymocytes. *N. Engl. J. Med.*, **292**, 775.

Maeda, H. (1979) Assay of proteolytic enzymes by the fluorescence polarization technique. *Analyt. Biochem.*, **92**, 222.

Maeda, H., Ishida, N., Kawauchi, H. & Tuzimara, K. (1969) Reaction of fluorescein-isothiocyanate with proteins and amino acids. I. Covalent and non-covalent binding. *J. Biochem.*, **65**, 777.

Maeda, H., Nakayama, M., Iwaoka, D. & Sato, T. (1979) Assay of angiotensin I by fluorescence polarization method. In *Kinins-II: Biochemistry, Pathophysiology and Clinical Aspects*. Ed. Fujii *et al.* New York: Plenum. p. 203.

Minowada, J., Ohkuma, T. & Moore, G. E. (1972) Rosette-forming human lymphoid cell lines. I. Establishment and evidence for origin of thymus-derived lymphocytes. *J. Natl Cancer Inst.*, **49**, 891.

Petitou, M., Tuy, F., Rosenfeld, C. & 5 others (1978) Decreased microviscosity of membrane lipids in leukemic cells: Two possible mechanisms. *Proc. Natl Acad. Sci. U.S.A.*, **75**, 2306.

Rosenthal, S., Canellos, G. P., Devita, V. & Gralnick, H. R. (1977) Characteristics of blast crisis in chronic granulocytic leukemia. *Blood*, **49**, 705.

Rotman, B. & Papermaster, B. W. (1966) Membrane properties of living mammalian cells as studied by enzymatic hydrolysis of fluorogenic esters. *Proc. Natl Acad. Sci. U.S.A.*, **55**, 132.

Shinitzky, M. & Inbar, M. (1974) Difference in microviscosity induced by different cholesterol levels in the surface membrane lipid layer of normal lymphocytes and malignant lymphoma cells. *J. Mol. Biol.*, **85**, 603.

Shinitzky, M. & Inbar, M. (1976) Microviscosity parameters and protein mobility in biological membranes. *Biochim. biophys. Acta*, **433**, 133.

Thomas, J. A., Buchsbaum, R. N., Zimniak, A. & Rackner, E. (1979) Intracellular pH measurement in Ehrlich ascites tumor cells utilizing spectroscopic probes generated *in situ*. *Biochemistry*, **18**, 2210.

Udkoff, R. & Norman, A. (1979) Polarization of fluorescein fluorescence in single cells. *J. Histochem. Cytochem.*, **27**, 49.

Yamane, I. & Tomioka, F. (1979) The concomitant effect of unsaturated fatty acid supplemented to medium on cellular growth and membrane fluidity of cultured cells. *Cell. Biol. Int. Rep.*, **3**, 515.